# Tapered Fiber Bragg Grating Fabry–Pérot Cavity for Sensitivity-Enhanced Strain Sensing

**DOI:** 10.3390/s26020581

**Published:** 2026-01-15

**Authors:** Jinchen Zhang, Chao Wang, Rui Dai, Yaqi Tang, Junhui Hu

**Affiliations:** 1 Guangxi Key Laboratory of Nuclear Physics and Technology, College of Physics Science and Technology, Guangxi Normal University, Guilin 541004, China; zhangjinchen@gxsfdx17.wecom.work; 2 Future Technology School, Shenzhen Technology University, Shenzhen 518118, China; drui12430@gmail.com (R.D.); 13856038134@163.com (Y.T.)

**Keywords:** fiber sensor, Fabry–Perot interferometer, fiber Bragg grating, strain

## Abstract

This paper presents a novel optical fiber axial strain sensor based on a Fabry–Perot interferometer (FPI) cavity incorporating Fiber Bragg Gratings (FBGs) and a tapered fiber, which has been experimentally validated. The sensor structure primarily consists of two identical FBGs with a bi-conical tapered fiber segment between them, achieving a strain sensitivity of 13.19 pm/με. This represents a 12-fold enhancement compared to conventional FBG-FPI, along with a resolution limit of 3.7 × 10^−4^ με. The proposed sensor offers notable advantages including low fabrication cost, compact structure, and excellent linearity, demonstrating significant potential for high-precision axial strain measurement applications.

## 1. Introduction

In recent years, optical fiber sensors have garnered significant attention due to their compact size, high sensitivity, immunity to electromagnetic interference, remote sensing capability, and multiplexing potential. Strain is a critical parameter in numerous applications, including structural health monitoring in civil engineering [1,2,3], aerospace and automotive industries [4,5,6], and biomedical and health monitoring [7,8,9]. Among various performance metrics, sensitivity is one of the most critical, driving extensive efforts to develop diverse sensing structures such as fiber Bragg grating (FBG) [10], long-period fiber grating (LPFG) [11], Mach–Zehnder interferometer (MZI) [12,13], Michelson interferometers (MI) [14,15], and Fabry–Pérot interferometer (FPI) [16,17] to meet application-specific requirements. In the early stages of optical fiber sensing development, research by Yun-Jiang Rao et al. [18] demonstrated that the strain sensitivity of fiber Bragg grating could reach 1.2 pm/με, which is fundamentally limited by the elasto-optic effect of the fiber material itself. Consequently, Yupeng Wang et al. [19] enhanced the strain sensitivity to 4.5 pm/με by employing chemical etch ing of FBG. In parallel, Fabry–Pérot interferometers, known for their narrow-linewidth transmission and reflection resonance peaks, have also been widely utilized for strain sensing due to their high wavelength resolution. For instance, Xinpu Zhang et al. [20] and He Wang et al. [21] developed an air-cavity FPI and a micro-bubble-based FPI, achieving strain sensitivities of 2.1 pm/με and 8.93 pm/με, respectively. With the maturation of FBG fabrication technology, FBG-FPI strain sensors have subsequently emerged. Chen Daru et al. [22] designed an FP cavity formed by two identical FBGs as mirrors. When the FBG-FP structure is under strain tuning, both the Bragg wavelength of the FBGs and the peak wavelength of the FP transmission band shift synchronously toward longer wavelengths, exhibiting an identical wavelength shift sensitivity of 1.1 pm/με. Due to the constraints of material properties, the FBG-FP structure does not yield a substantial enhancement in strain sensitivity. In this paper, the tapered FBG-FP structure proposed in this work leverages the strain concentration effect to achieve a remarkable enhancement in strain sensitivity while preserving the high-resolution advantage inherent to FP cavities. This dual advantage positions the tapered FBG-FPI as a promising candidate for precision strain measurement applications.

This study demonstrates a strain sensing system that utilizes a narrow-linewidth fiber laser to interrogate an FBG-FP structure. By implementing a servo-controlled closed-loop feedback locking scheme, the system converts intensity variations at a specific point on the resonance slope—caused by strain-induced wavelength shifts—into measurable feedback voltage signals. This approach offers the advantages of high sensitivity and rapid response. The final results indicate that the sensor achieves a strain sensitivity of 13.19 pm/με and a resolution limit of 3.7 × 10^−4^ με, representing an approximately 12-fold enhancement compared to conventional FBG-FPI sensors.

## 2. Sensing Principle

### 2.1. Operating Principle

The Fabry–Perot cavity is formed by two Fiber Bragg Gratings acting as mirrors, in which light undergoes multiple reflections, resulting in optical interference. The resonance condition is satisfied when the round-trip phase difference is an integer multiple of 2π [23].

(1) $$2n_{eff}L=m\lambda$$

Here, *n_eff_* is the effective refractive index of the fiber, *L* is the length of the FP cavity, *m* is the resonance order (m = integer), and *λ* is the resonance wavelength. When the FP cavity is subjected to strain, its physical length changes, resulting in a corresponding shift in the resonance wavelength.

When the FP cavity is a non-uniform cavity, the resonance condition is given by:

(2) $$\int_{0}^{L_{c}}n_{eff}(z)dz=\frac{m\lambda_{m}}{2}$$

A total differential is performed on the resonance condition:

(3) $$\begin{array}{l} \int_{0}^{L_{c}}\Delta n_{eff}\left(z\right)dz+\int_{0}^{L_{c}}n_{eff}\left(z\right)\Delta \left(dz\right)=\frac{m}{2}\Delta \lambda_{m} \end{array}$$

The relationship between the local strain $\begin{array}{l} \frac{\Delta dz}{dz} \end{array}$ and the global average strain *ε* is as follows:

(4) $$\begin{array}{l} \int_{0}^{L_{c}}\Delta n_{eff}\left(z\right)dz+\varepsilon \int_{0}^{L_{c}}n_{eff}\left(z\right)M\left(z\right)dz=\frac{m}{2}\Delta \lambda_{m} \end{array}$$

The variation in the effective refractive index is primarily induced by the elasto-optic effect, as described by the following relation:

(5) $$\begin{array}{c} \Delta n_{neff}(z)=-P_{e}\cdot n_{eff}(z)\cdot M(z)\varepsilon \end{array}$$

Substituting Equation (5) into Equation (4), combining and simplifying yields:

(6) $$\begin{array}{l} \varepsilon \int_{0}^{L_{c}}\left[\left(1-P_{\varepsilon }\right)n_{eff}\left(z\right)\right]M\left(z\right)dz=\frac{m}{2}\Delta \lambda_{m} \end{array}$$

where *Lc* is the resonator length, *Pε* is the elasto-optic coefficient and *M* is the local strain amplification factor. Finally, the variation in the resonant wavelength with strain at constant temperature can be expressed as

(7) $$\frac{\Delta \lambda_{m}}{\lambda_{m}}=\varepsilon \cdot \frac{\int_{0}^{L_{c}}\left(1-P_{\varepsilon }\right)n_{eff}\left(z\right)M\left(z\right)dz}{\int_{0}^{L_{c}}n_{eff}\left(z\right)dz}$$

The local strain amplification factor *M* can be derived through mechanical analysis. Given that the fiber is a continuous body, the axial force is identical across any cross-section. According to Hooke’s law, the strain in different sections of the same fiber is inversely proportional to their respective cross-sectional areas. Hence, the relationship between the globally averaged strain and the local strain, expressed by the amplification factor *M*, is given by [24]

(8) $$M_{i}=\frac{\varepsilon_{i}}{\varepsilon }=\frac{L_{b}}{{\left(d_{i}\right)}^{2}\sum_{i=1}^{n}\frac{L_{i}}{{\left(d_{i}\right)}^{2}}}$$

Here, *L_b_* denotes the total length of the strain measurement substrate, while *ε_i_* and *d_i_* represent the strain and diameter at the corresponding position, respectively. When the substrate length *L_b_* = 53 cm, the single-mode fiber diameter *D* = 125 μm, the waist diameter *d* = 35 μm, and the waist length and transition length are 1 cm and 2 cm, respectively, substituting these parameters into the formula yields a strain amplification factor *M* of 9.55.

### 2.2. Simulation of Strain Concentration in Tapered Fiber

**Figure 1:**
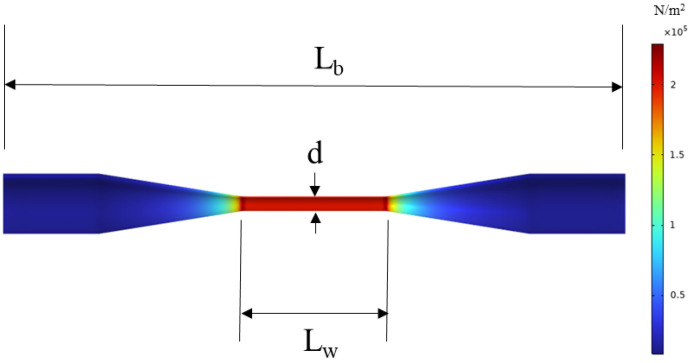
Stress Distribution in the Tapered Fiber Structure.

The mechanical response of the tapered fiber under applied strain was analyzed using the Solid Mechanics module in COMSOL Multiphysics. The configuration of the mechanical model is illustrated in Figure 1, with an initial waist diameter of *d* = 35 μm and a waist length of *L_w_* = 0.3 mm. In the simulation, the left end face was fixed, while a displacement was applied to the right end face to induce a strain of 1 µε in the tapered fiber. As shown in Figure 1, the stress is predominantly concentrated in the waist region and is uniformly distributed along sections with constant diameter. This observed stress distribution confirms the critical role of the waist region and justifies further investigation into the influence of waist diameter on the overall mechanical behavior of the tapered fiber.

**Figure 2:**
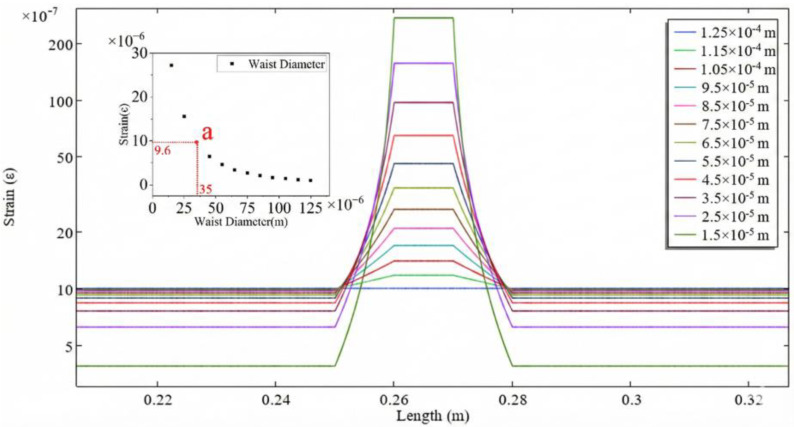
Effect of different waist diameter on strain (a: Actual Sample Parameters).

**Figure 3:**
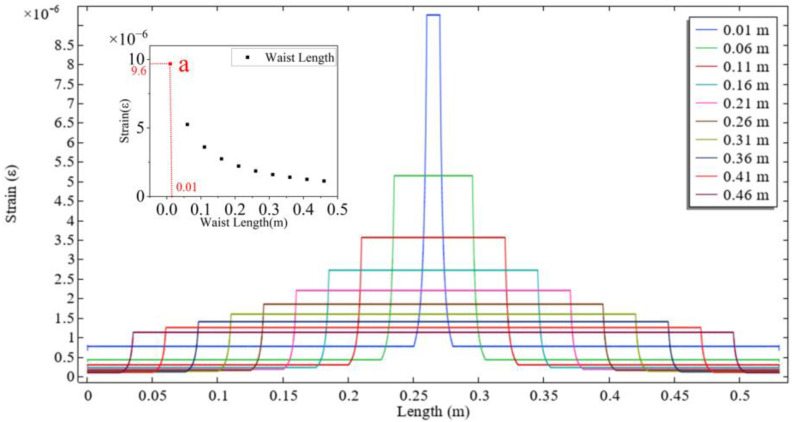
Effect of different waist length on strain (a: Actual Sample Parameters).

The optical fiber length was fixed at *L* = 53 cm and the waist length at *L_w_* = 1 cm, while the waist diameter was reduced from 125 μm to 15 μm. As shown in Figure 2, the strain in the waist region increases as the waist diameter decreases. The use of geometry merging during the modeling process introduces non-smooth strain response artifacts at the junction between the transition region and the waist region. Theoretically, a smaller diameter leads to higher strain sensitivity of the sensor. The influence of waist length was further investigated. With the fiber length fixed at *L* = 53 cm and the waist diameter at *d* = 35 *μ*m, the waist length was increased from 0.01 m to 0.46 m. As illustrated in Figure 3, the strain in the waist region decreases with increasing waist length. Therefore, minimizing the waist length can further enhance the strain sensitivity of the sensor.

To balance the trade-offs between mechanical strength, fabrication reproducibility, and the need to avoid the emergence of an evanescent field, the final fabrication parameters were selected at point a in both Figure 2 and Figure 3. At this operating point, the strain in the waist region is 9.6 times that in the untapered fiber, demonstrating close agreement with the theoretical value of 9.55 obtained from the formula. Furthermore, the fracture limit of the entire fiber under this waist diameter reaches 4050 µε.

## 3. Fabrication

The tapered FBG-FP structure consists of two identical FBGs and a section of tapered fiber (Corning Incorporated, Corning, NY, USA, SMF-28e). Both FBGs have a length of 2 mm, a reflectivity of approximately 87%, and a center wavelength around 1550 nm. The spacing between the two FBGs is approximately 13 cm. The diameter of the tapered fiber region is about 35 μm, with a length of 1 cm.

**Figure 4:**
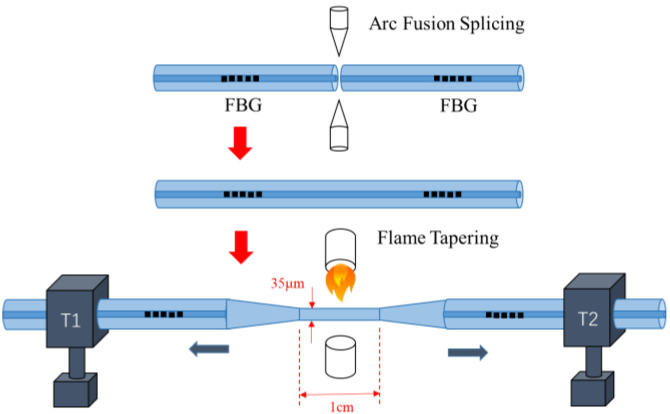
Fabrication process of the tapped FBG-FP.

The fabrication process of the sample is show in Figure 4. First, two FBG samples were prepared. A section of single-mode fiber of specified length was retained on one side of each FBG. The coating of this single-mode fiber section was carefully stripped using a blade to avoid damaging the FBG structure, and the stripped fiber was subsequently cleaned with alcohol. The fiber end face was then cleaved using a fiber cleaver (FITEL, Chiyoda-ku, Tokyo, Japan, S326) while controlling the length of the single-mode fiber to achieve the desired FBG-FP cavity length. The two prepared FBGs were then fusion spliced together using a fusion splicer (BEIJING W&F, Beijing, China, WF680). Finally, the sample was placed in an oxyhydrogen flame tapering system (Shandong Kaipule Photoelectric Technology, Tai’an, China, AFBT-8000) and secured on translation stages. During the heating process, the stages moved simultaneously in opposite directions at a speed of 100 μm/s to form a biconical taper structure.

## 4. Result and Discussion

### 4.1. System Description

**Figure 5:**
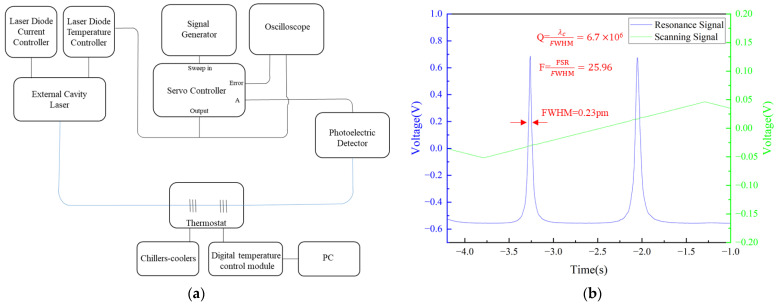
(**a**) Scanning System Diagram (blue lines: fiber optic cables, black lines: electrical wires); (**b**) resonance spectrum of the tapered FBG-FPI.

When the Fabry–Perot cavity reaches a certain length, the distance between two adjacent resonant peaks becomes smaller than the wavelength resolution of the Optical Spectrum Analyzer (OSA), making it impossible to resolve the specific features of the resonance peaks from the OSA. To overcome this limitation, an External Cavity Diode Laser (THORLABS, Newton, NJ, USA, SFL1550P) was employed to scan the vicinity of the center wavelength of a 87% Fiber Bragg Grating to observe the resonance peaks. A triangular wave was applied to the temperature controller (THORLABS, Newton, NJ, USA, TED200C) via a signal generator (KEYSIGHT, Santa Rosa, CA, USA, 33500B) to modulate the laser wavelength, with a scanning amplitude of 80 mV and a scanning frequency of 200 mHz. The experimental setup is shown in Figure 5a.

The temperature and current tuning coefficients of the laser are 0.083 nm/kΩ and 0.0005 nm/mA, respectively, enabling the conversion of the time interval between resonance peaks into the corresponding wavelength difference. As shown in Figure 5b, the measured time interval between the two resonance peaks was approximately 1.21 s, corresponding to a Free Spectral Range (FSR) of 5.97 pm. The full width at half maximum (FWHM) time difference in the FBG-FPI resonance was about 0.046 s, corresponding to a wavelength difference of 0.23 pm. The resonant cavity exhibits a central wavelength of 1550 nm, a finesse of 25.96, and a Q-factor of 6.7 × 10^6^.

The resonant side lock of a high-speed servo controller is a feedback control technology commonly used for laser frequency or intensity stability. Its core principle is to use the slope of the optical resonance curve as an error signal to lock the system on a slope near the resonant point through the servo controller.

**Figure 6:**
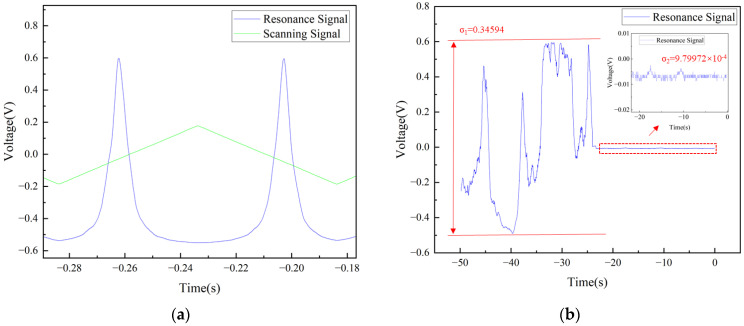
(**a**) Electronically controlled scanning; (**b**) signal comparison before and after locking.

The laser is scanned using a current controller (THORLABS, Newton, NJ, USA, LDC202C) with a small tuning range and high precision, with a scanning amplitude of 300 mV and a scanning frequency of 10 Hz. The photodetector (Newport, Irvine, CA, USA, 2011FC-M) converted the optical signal transmitted through the sensor into an electrical signal, which was then fed into port A of the servo controller (NEW FOCUS, San Jose, CA, USA, LB1005). The scanning amplitude was adjusted to display a complete resonance peak on the oscilloscope (KEYSIGHT, Santa Rosa, CA, USA, DSOX2024A). Subsequently, fine adjustments were made to both the scan center and input offset to position the target lock point near the zero point of the error signal, as illustrated in Figure 6a. This lock point typically corresponds to the region of maximum slope on the resonance flank, specifically at the half-maximum points of the resonance peak. As demonstrated in Figure 6b, when scanning was halted, significant fluctuations in signal intensity were observed at this point, which stabilized near the zero point after lock acquisition. By continuously monitoring intensity variations at this location, the servo controller generated a corresponding error signal. This signal was processed through a proportional-integral (P-I) filter, and the resulting control signal was used to drive the laser current controller, thereby maintaining system stability at the lock point.

### 4.2. Strain Testing

In the slope locking scheme, the system signal is extracted by monitoring the feedback voltage required to maintain the lock. Applied strain alters the resonance condition of the FBG-FP cavity, thus inducing changes in the feedback voltage. These voltage variations are subsequently converted into wavelength shifts using the laser’s current tuning coefficient, establishing a proportional relationship between wavelength change and applied strain.

**Figure 7:**
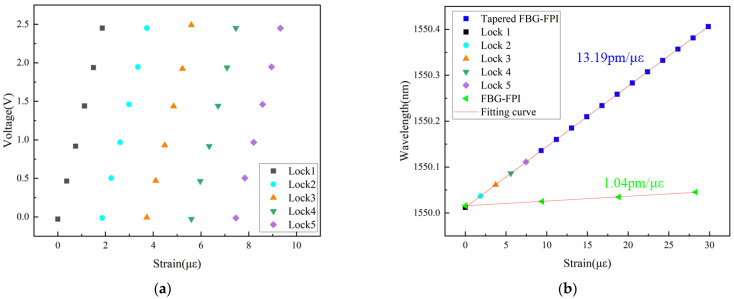
(**a**) Relationship between feedback voltage and strain under different locking instances. (**b**) Linear fit between strain and wavelength.

The strain applied to the tapered FBG-FPI was controlled by a high-precision three-axis translation stage, with an initial distance of 0.53 m between the two stages and a displacement step of 0.2 μm. To mitigate the influence of ambient temperature fluctuations on the resonance peaks, the tapered FBG-FPI was placed within a temperature-controlled chamber maintained at 25 °C by a water bath throughout the experiment. Since the single-frequency tuning range of the laser directly limits the sensor’s continuous measurement range, the system loses its lock state when the applied strain exceeds this range. At this point, the system can be re-locked to an adjacent resonance peak, and the measurement continues. This process is repeated sequentially until reaching the sensor’s maximum operating range of 1350 με. Since the locking process for each resonance peak is independent, the wavelength shifts from different lock cycles can be accumulated to obtain the total wavelength shift induced by the overall applied strain, thereby achieving an extended measurement range. The relationship between feedback voltage and applied strain over five consecutive lock cycles is shown in Figure 7a. Notably, the voltage change for an identical strain step remains consistent across different lock cycles, confirming the feasibility of determining the final wavelength shift by summing the individual voltage changes. The strain application process was continuous. The total voltage change recorded during each lock cycle was selected, and the cumulative wavelength shift was reconstructed by successively summing these voltage variations converted via the laser’s tuning coefficient. Thus, after *n* lock cycles, the total wavelength shift corresponding to the applied strain is given by

(9) $$\lambda_{\varepsilon }=\lambda_{0}+0.01\cdot \sum_{i=1}^{n}V\left(n\right)$$

where *λ*_0_ is the central wavelength at the initial lock, and *V*(*n*) denotes the voltage variation induced by the strain after the *n*-th lock acquisition, with a tuning coefficient of 0.01 nm/V.

As shown in Figure 7b, a total of 17 lock cycles were performed. The total voltage change for each cycle was taken, and the corresponding wavelength was calculated using Equation (9). Linear fitting of the resulting data yielded a strain sensitivity of 13.19 pm/με for the tapered FBG-FPI. This represents an approximately 12-fold enhancement compared to the sensitivity of the pre-tapered FBG-FPI (1.04 pm/με). The experimental amplification factor exceeded the simulated value of 9.6, which is attributed to the fact that strain-induced changes in the effective refractive index were not accounted for in the simulation. With a laser current controller precision of 0.01 mA, the tapered FBG-FPI strain sensing system achieves a resolution limit of 3.7 × 10^−4^ με.

**Figure 8:**
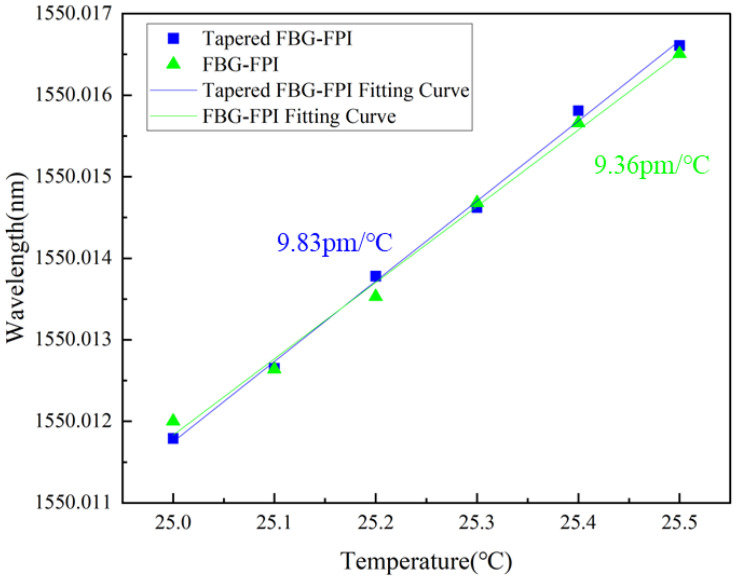
Linear fit between temperature and wavelength.

The temperature response experiment was performed by heating a temperature-controlled chamber using a water bath, raising the temperature from 25 °C to 25.5 °C in steps of 0.1 °C. The corresponding changes in feedback voltage and temperature were recorded and then converted into the relationship between wavelength shift and temperature. Linear fitting of the data yields temperature sensitivities of 9.83 pm/°C and 9.36 pm/°C for the tapered FBG-FPI and the conventional FBG-FPI, respectively, as shown in Figure 8.

Unlike the strain sensitivity, the temperature sensitivities of the tapered and conventional FBG-FPI are nearly identical. This similarity arises because both structures are fabricated from the same optical fiber material, which possesses identical thermal expansion and thermo-optic coefficients. Furthermore, the temperature distribution along the fiber is uniform during the test, resulting in essentially the same temperature response for both configurations. The calculated temperature cross-sensitivity of the tapered FBG-FPI is 0.74 µε/°C. Therefore, to eliminate temperature-induced interference during strain sensing experiments, the sample can be placed in a temperature-stabilized chamber (Table 1).

## 5. Conclusions

In summary, this work presents and experimentally validates a strain sensing system based on a tapered FBG-FPI, achieving a strain sensitivity of 13.19 pm/µε and a resolution of 3.7 × 10^−4^ µε. This represents a significant enhancement in strain sensitivity over the conventional FBG-FPI. By adopting a slope-locking scheme, strain-induced resonant wavelength shifts are precisely detected via feedback voltage measurements. This method can be extended to high-resolution sensing of other parameters, such as dynamic strain, refractive index, pressure, and acoustic waves. In future work, employing a laser with a wider tuning range would increase the measurement span per single lock, thereby reducing post-processing complexity and further advancing the development of high-precision, miniaturized sensing technologies. Moreover, the all-fiber architecture of the sensor offers inherent advantages of compactness, immunity to electromagnetic interference, and potential high-temperature endurance. It demonstrates considerable application promise in cutting-edge fields that demand high accuracy and reliability, such as micro-strain monitoring of high-temperature components in aerospace engines, mechanical assessment of biomedical implants, performance testing of micro/nano-electromechanical systems, and safety monitoring in extreme environments of nuclear facilities.

## Figures and Tables

**Table 1:** Performance comparison of optical fiber strain sensors.

Structures	Axial Strain Sensitivity	Resolution	Linear Range of Detection	Refs
FBG	1.2 pm/με	16.6 με	0–1000 με	[18]
Etched FBG	4.5 pm/με	4.4 με	0–1000 με	[19]
Air-cavity FPI	2.1 pm/με	9.5 με	0–2000 με	[20]
Micro-bubble based FP	8.93 pm/με			[21]
Parallel FPI with Vernier effect	43.2 pm/με	0.231 µε		[25]
FBG-FPI	1.1 pm/με	0.11 με		[22]
Air cavity FP cascaded FBG	5 pm/µε			[26]
Spheroidal-Cavity-Overlapped FBG	3.76 pm/µε		0–500 με	[27]
Adiabatic Taper FBG-FP	2.32 pm/με		0–3400 με	[28]
Rounded rectangular air cavity FPI	8 pm/µε		0–1200 µε	[29]
asymmetric tapered air microbubble FPI	15.89 pm/µε		0–1200 µε	[30]
Tapered FBG-FPI	13.38 pm/με	3.8 × 10^−4^ με	0–1350 με	This work

## Data Availability

Data are contained within this article.
